# Supplementary material for: Application of Spectroscopic Methods for the Identification of Superoxide Dismutases in Cyanobacteria
Source: Int J Mol Sci. 2022 Nov 10;23(22):13819. doi: 10.3390/ijms232213819 (PMC9692673; doi:10.3390/ijms232213819)
Supplement: Supplementary file 1 [file ijms-23-13819-s001.zip › ijms-1980437-supplementary.pdf]

Supplementary materials

Application of spectroscopic methods for the identification of superoxide dismutases in cyanobacteria

Monika Kula-Maximenko [1], Kamil Jan Zieliński [1], Joanna Depciuch [2], Janusz Lekki [2], Marcin Niemiec [3] and Ireneusz Ślesak [1,*]

[1] The Franciszek Górski Institute of Plant Physiology, Polish Academy of Sciences, Niezapominajek 21, 30-239 Krakow, Poland
[2] Institute of Nuclear Physics, Polish Academy of Sciences, 31-342 Krakow, Poland
[3] Department of Agricultural and Environmental Chemistry, University of Agriculture in Krakow, 31-120 Krakow, Poland
* Correspondence: i.slesak@ifr-pan.edu.pl; Tel.: +48-124-251-833

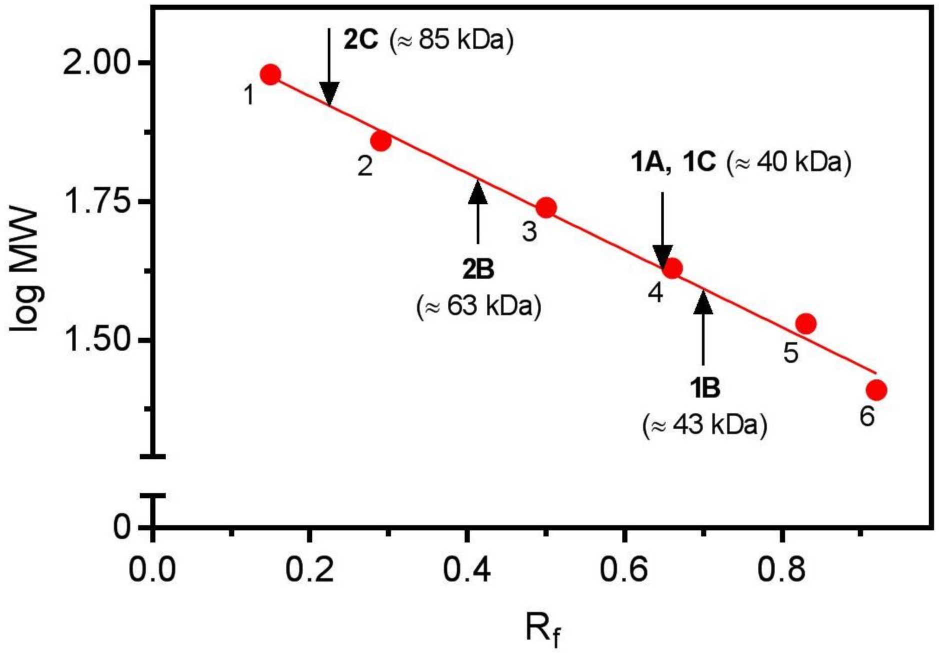

Figure S1. Determining the molecular weight (MW) of SODs from Figure 2. (the main text of the article). Arrows indicate the estimated MW (kDa) for each SOD band: 1A, 1B, 1C, 2B, and 2C. A standard curve of the log MW vs. R_f was generated using the PageRuler™ Prestained Protein Ladder (Thermo Scientific) from Figure 2 (the main text of the article). The strong relationship ($R^2 = 0.9904$, $y = -0.6952x + 2.08$, $P < 10^{-4}$) between the proteins' MW and the relative migration distance (R_f) demonstrates reliability in predicting MW. R_f is defined as the migration distance of the protein through the gel divided by the migration distance of the dye front. The numbers near calibration points represent marker proteins from the PageRuler™ Prestained Protein Ladder: 1. 95 kDa, 2. 72 kDa, 3. 55 kDa, 4. 43 kDa, 5. 34 kDa, 6. 26 kDa.

Table S1. Parameters, i.e., log MW and R_f values used for preparation the curve from Figure S1.

**No.**	**MW [kDa]**	**log MW**	**R_f**
1	95	1.98	0.15
2	72	1.86	0.29
3	55	1.74	0.50
4	43	1.63	0.66
5	34	1.53	0.83
6	26	1.41	0.92

Table S2. MW of SODs determined based on the curve from Figure S1.

**SOD activity bands**	**R_f**	**log MW[a]**	**Estimated MW [kDa][b]**
1A (FeSOD1)	0.7	1.6	~ 40 (39.8)
1B (FeSOD1)	0.65	1.63	~ 43 (42.6)
1C (FeSOD1)	0.7	1.6	~ 40 (39.8)
2B (FeSOD2)	0.41	1.8	~ 63 (63.09(
2C (FeSOD2)	0.22	1.93	~ 85 (85.1)

[a]Values calculated based on the equation of the curve from Fig. 1S, i.e. y = -0.6952x + 2.08

[b]Calculated based on MW = 10^y

Notes S1.

Sequenced 16S rRNA fragments for *Geitlerinema* sp. ZHR1A and *G. violaceus* CCALA979

```
> Geitlerinema sp. ZHR1A, 16S rRNA  (fragment) obtained using CYA 359F and
CYA 781R
TGTGGGAATTTTCCGCAATGGGCGAAAGCCTGACGGAGCAATACCGCGTGAGGGACGAAGGCCTGTGGGTTGTAA
ACCTCTTTTCTCAGGGAAGAAGATCTGACGGTACCTGAGGAATCAGCATCGGCTAACTCCGTGCCAGCAGCCGCG
GTAAGACGGAGGATGCAAGCGTTATCCGGAATTATTGGGCGTAAAGCGTCCGCAGGCGGTCTCGTAAGTCTGTCT
TTAAAGCGTGGAGCTTAACTCCATAAAGGGGATGGAAACTGCGAGACTAGAGGTAGGTAGGGGTAGAAGGAATTC
CCAGTGTAGCGGTGAAATGCGTAGATATTGGGAAGAACACCAGCAGCGAAGGCGTTCTACTGGACCAAACCTGAC
GCTCATGGACGAAAGCTAGGGGAGCGAAWGGGATTAGATACCCCWGTAGTCAA

> Gloeobacter violaceus CCALA 979, 16S rRNA (fragment) obtained using CYA
359F and CYA 781R
TGTGTGAATTTTCCGCAATGGGCGAAAGCCTGACGGAGCAACGCCGCGTGGGTGATGAAGGCCCGTGGGTCGTAA
AGCCCTTTTGTCAGGGACGAAACCTGACGGTACCTGACGAATAAGCATCGGCTAACTCCGTGCCAGCAGCCGCGG
TAATACGGAGGATGCAAGCGTTATCCGGAATTATTGGGCGTAAAGCGTACGTAGGCGGCCAGCCAAGTCTGGGGT
CAAAGCCCGCAGCCTAACTGCGGAAAGGCCCTGGAAACTGGTTGGCTAGAGTGCGGTAGGGGCAAGGGGAATTCC
CGGTGTAGCGGTGAAATGTGGAGATATCGGGAAGAACACCAGCGGCGAAAGCGCCTTGCTGGACCGCAACTGACG
CTGAGGTACGAAAGCCAGGGGAGCAAAWGGGATTAGATACCCCWGTAGTACA
```

Y: C/T

W: A/T

Notes S2.

All 16S rRNA sequences used for reconstruction of the phylogenetic tree

>ENA|BA000045.2:1571231..1572715:rRNA|BA000045.2:1571231..1572715:rRNA.2 Gloeobacter violaceus PCC 7421 16S rRNA
AAAATGGAGAGTTTGATCCTGGCTCAGGATGAACGCTGGCGGCGTGCTTAACACATGCAAGTCGAACGAGATCTTCGGATCGAGTGGCGGACGG
GTGAGTAACACGTGGGAATCTGCCCTCAGGAGGGGGATAACGGCCGGAAACGGCCGCTAATACCCCATATGCCGCAAGGTGAAATGAATTTCGC
CTGAGGATGAGCCCGCGTCTGATTAGCTAGTTGGTGGGGTAATGGCCTACCAAGGCTACGATCAGTAGCTGGTCTGAGAGGATGATCAGCCACA
CTGGGACTGAGACACGGCCCAGACTCCTACGGGAGGCAGCAGTGGGGAATTTTCCGCAATGGGCGAAAGCCTGACGGAGCAACGCCGCGTGGGT
GATGAAGGCCCGTGGGTCGTAAAGCCCTTTTGTCAGGGACGAAACCTGACGGTACCTGACGAATAAGCATCGGCTAACTCCGTGCCAGCAGCCG
CGGTAATACGGAGGATGCAAGCGTTATCCGGAATTATTGGGCGTAAAGCGTACGTAGGCGGCCAGCCAAGTCTGGGGTCAAAGCCCGCAGCCTA
ACTGCGGAAAGGCCCTGGAAACTGGTTGGCTAGAGTGCGGTAGGGGCAAGGGGAATTCCCGGTGTAGCGGTGAAATGTGGAGATATCGGGAAGA
ACACCAGCGGCGAAAGCGCCTTGCTGGACCGCAACTGACGCTGAGGTACGAAAGCCAGGGGAGCAAATGGGATTAGATACCCCAGTAGTCCTGG
CCGTAAACGATGGACACTAGGTGTTGCGCGTATCGACCCGTGCAGTGCCGTAGCTAACGCGTTAAGTGTCCCGCCTGGGGAGTACGCACGCAAG
TGTGAAACTCAAAGGAATTGACGGGGGCCCGCACAAGCGGTGGAGCATGTGGTTTAATTCGATGCAACGCGAAGAACCTTACCAGGGCTTGACA
TGTCGCGAATCTCGCGGAAACGTGGGAGTGCCTTCGGGAACGCGAACACAGGTGCTGCATGGCTGTCGTCAGCTCGTGTCGTGAGATGTTGGGT
TAAGTCCCGCAACGAGCGCAACCCTCGTCGTCAGTTGCCATCATTCAGTTGGGCACTCTGGCGAGACTGCCGGTGACAAACCGGAGGAAGGTGG
GGATGACGTCAAGTCAGCATGGCTCTTACGTCCTGGGCTACACACGTGCTACAATGGTTCGGATAAAGGGTCGCAATCTCGCGAGGGGGAGCCA
ATCCCATAAACCGAATCTCAGTTCGGATTGCAGGCTGCAACTCGCCTGCATGAAGTCGGAATCGCTAGTAATCGCAGGTCAGCATACTGCGGTG
AATACGTTCCCGGGCCTTGTACACACCGCCCGTCACACCATGGAAGCTGGTCATGCCCGAAGTCGGTATTCTAACCGCAAGGAGGGAGCCGCCG
AAGGCGGGGCCGGTGACTGGGGTGAAGTCGTAACAAGGTAGCCGTACCGGAAGGTGCGGCTGGATCACCTCCTTT

>ENA|CP003587.1:2731683..2733167:rRNA|CP003587.1:2731683..2733167:rRNA.1 Gloeobacter kilaueensis JS1 16S ribosomal RNA
AAAATGGAGAGTTTGATCCTGGCTCAGGATGAACGCTGGCGGCGTGCTTAACACATGCAAGTCGAACGAGATCTTCGGATCTAGTGGCGGACGG
GTGAGTAACACGTGGGAATCTGCCCTCAGGAGGGGGATAACATTCGGAAACGAGTGCTAATACCCCATATGCCGCAAGGTGAAATGAATTTCGC
CTGAGGATGAGCCCGCGTCTGATTAGCTAGTTGGTGGGGTAATGGCCTACCAAGGCGACGATCAGTAGCTGGTCTGAGAGGATGATCAGCCACA
CTGGGACTGAGACACGGCCCAGACTCCTACGGGAGGCAGCAGTGGGGAATTTTCCGCAATGGGCGAAAGCCTGACGGAGCAACGCCGCGTGGGT
GATGAAGGCCCGTGGGTCGTAAAACCCTTTTGTCAGGGACGAAACCTGACGGTACCTGACGAATAAGCATCGGCTAACTCCGTGCCAGCAGCCG
CGGTAATACGGAGGATGCAAGCGTTATCCGGAATTATTGGGCGTAAAGCGTACGTAGGTGGCCAGCCAAGTCTGGGGTCAAAGCCCGCAGCCTA
ACTGCGGAAAGGCCCTGGAAACTGGTTGGCTAGAGTGCGGTAGGGGCAAGGGGAATTCCCGGTGTAGCGGTGAAATGTGGAGATATCGGGAAGA
ACACCAGCGGCGAAAGCGCCTTGCTGGACCGCAACTGACGCTGAGGTACGAAAGCCAGGGGAGCAAATGGGATTAGATACCCCAGTAGTCCTGG
CCGTAAACGTTGGACACTAGGTGTTGCGCGTATCGACCCGTGCAGTGCCGTAGCTAACGCGTTAAGTGTCCCGCCTGGGGAGTACGCACGCAAG
TGTGAAACTCAAAGGAATTGACGGGGGCCCGCACAAGCGGTGGAGCATGTGGTTTAATTCGATGCAACGCGAAGAACCTTACCAGGGCTTGACA
TGTCGCGAACCTCGCGGAAACGTGGGGGTGCCTTCGGGAACGCGAACACAGGTGCTGCATGGCTGTCGTCAGCTCGTGTCGTGAGATGTTGGGT
TAAGTCCCGCAACGAGCGCAACCCTCGTCGTCAGTTGCCATCATTCAGTTGGGCACTCTGGCGAGACTGCCGGTGACAAACCGGAGGAAGGTGG
GGATGACGTCAAGTCAGCATGGCTCTTACGTCCTGGGCTACACACGTGCTACAATGGTTCGGATAAAGGGCTGCAATCCCGCGAGGGGGAGCCA
ATCCCATAAACCGAATCTCAGTTCGGATTGTAGGCTGCAACTCGCCTACATGAAGTCGGAATCGCTAGTAATCGCAGGTCAGCATACTGCGGTG
AATACGTTCCCGGGCCTTGTACACACCGCCCGTCACACCATGGGAGCTGGTCATGCCCGAAGTCGGTATCCTAACCGCAAGGAGGGAGCCGCCG
AAGGCGGGGCCGGTGACTGGGGTGAAGTCGTAACAAGGTAGCCGTACCGGAAGGTGCGGCTGGATCACCTCCTTT

> Gloeobacter violaceus CCALA 979, 16S rRNA (fragment) obtained using CYA 359F and CYA 781R
TGTGTGAATTTTCCGCAATGGGCGAAAGCCTGACGGAGCAACGCCGCGTGGGTGATGAAGGCCCGTGGGTCGTAAAGCCCTTTTGTCAGGG
ACGAAACCTGACGGTACCTGACGAATAAGCATCGGCTAACTCCGTGCCAGCAGCCGCGGTAATACGGAGGATGCAAGCGTTATCCGGAATT
ATTGGGCGTAAAGCGTACGTAGGCGGCCAGCCAAGTCTGGGGTCAAAGCCCGCAGCCTAACTGCGGAAAGGCCCTGGAAACTGGTTGGCTA
GAGTGCGGTAGGGGCAAGGGGAATTCCCGGTGTAGCGGTGAAATGTGGAGATATCGGGAAGAACACCAGCGGCGAAAGCGCCTTGCTGGAC
CGCAACTGACGCTGAGGTACGAAAGCCAGGGGAGCAAAWGGGATTAGATACCCCWGTAGTACA

>ENA|BA000039.2:2335243..2336733:rRNA|BA000039.2:2335243..2336733:rRNA.2 Thermosynechococcus elongatus BP-1 16S rRNA
AAAATGGAGAGTTTGATCCTGGCTCAGGATGAACGCTGGCGGTCTGCTTAACACATGCAAGTCGAACGAGCACTTCGGTGCTAGTGGCGGACGG
GTGAGTAACACGTGAGAATCTACCCTCAGGAGGGGGATAACGGCTGGAAACGGCCGCTAATACCCCATATGCCGCAAGGTGAAATCTTTTTTGG
CCTGGGGATGAGCTCGCGGTGGATTAGCTAGTTGGTGGGGTAATGGCCTACCAAGGCGACGATCCATAGCTGGTCTGAGAGGATGATCAGCCAC
ACTGGGACTGAGACACGGCCCAGACTCCTACGGGAGGCAGCAGTGGGGAATTTTCCGCAATGGGCGAAAGCCTGACGGAGCAAGACCGCGTGAG
GGATGAAGGCCTTTGGGTTGTAAACCTCTTTTCTCAGGGAAGAACACAATGACGGTACCTGAGGAATAAGCCTCGGCTAACTCCGTGCCAGCAG
CCGCGGTAAGACGGAGGAGGCAAGCGTTATCCGGAATTATTGGGCGTAAAGCGTCCGCAGGTGGCTTTCCAAGTCTGCCGTCAAAGCCCGAGGC
TTAACCTCGGATCGGCGGTGGAAACTGGATCGCTAGAGTGCGTCAGGGGTAGGGGGAATTCCCGGTGTAGCGGTGAAATGCGTAGATATCGGGA
AGAACACCAGTGACGAAAGTGCCCTACTGGGACGTTACTGACACTCATGGACGAAAGCTAGGGGAGCGAAAGGGATTAGATACCCCTGTAGTCC
TAGCCGTAAACGATGGACACTAGGTGTTGCTGGTATCCACTCCAGCAGTGCCGTAGCCAACGCGTTAAGTGTCCCGCCTGGGGAGTACGCACGC
AAGTGTGAAACTCAAAGGAATTGACGGGGGCCCGCACAAGCGGTGGAGTATGTGGTTTAATTCGATGCAACGCGAAGAACCTTACCAGGGCTTG
ACATGTCGCGAATCCCGCGGAAACGTGGGAGTGCCTTCGGGAACGCGAACACAGGTGGTGCATGGCTGTCGTCAGCTCGTGTCGTGAGATGTTG
GGTTAAGTCCCGCAACGAGCGCAACCCTCGTCCTTAGTTGCCAGCATTCAGTTGGGCACTCTAGGGAGACTGCCGGTGACAAACCGGAGGAAGG
TGGGGATGACGTCAAGTCATCATGCCCCTTACGTCCTGGGCTACACACGTACTACAATGCTGTGGACAAAGAGTTGCCAACCCGCGAGGGCGAG
CTAATCTCTTAAACCACGGCTCAGTTCAGATTGCAGGCTGCAACTCGCCTGCATGAAGGCGGAATCGCTAGTAATCGCAGGTCAGCATACTGCG
GTGAATACGTTCCCGGGCCTTGTACACACCGCCCGTCACACCATGGGAGTTGGCCATGCCCGAAGTCGTTACTCTAACCCGCAAGGGAGGGGGA
CGCCTAAGGCAGGGCTGATGACTGGGGTGAAGTCGTAACAAGGTAGCCGTACCGGAAGGTGTGGCTGGATCACCTCCTTTT

>ENA|AP012205.1:2451003..2452491:rRNA|AP012205.1:2451003..2452491:rRNA.1 Synechocystis sp. PCC 6803 16S ribosomal RNA
ACAATGGAGAGTTTGATCCTGGCTCAGGATGAACGCTGGCGGTATGCCTAACACATGCAAGTCGAACGGAGTTCTTCGGAACTTAGTGGCGGAC
GGGTGAGTAACACGTGAGAACCTACCTTCAGAATGGGGACAACAGTTGGAAACGACTGCTAATACCCAATGTGCCGAAAGGTGAAAGATTTATC
GTCTGAAGATGGGCTCGCGTCTGATTAGCTAGATGGTGGGGTAAGAGCCTACCATGGCAACGATCAGTAGCTGGTCTGAGAGGATGAGCAGCCA
CACTGGGACTGAGACACGGCCCAGACTCCTACGGGAGGCAGCAGTGGGGAATTTTCCGCAATGGGCGAAAGCCTGACGGAGCAATACCGCGTGA
GGGAGGAAGGTCCTTGGATTGTAAACCTCTTTTATCAGGGAAGAAGTTCTGACGGTACCTGATGAATAAGCATCGGCTAACTCCGTGCCAGCAG

CCGCGGTAATACGGAGGATGCAAGCGTTATCCGGAATTATTGGGCGTAAAGCGTCCGTAGGTGGTTATGCAAGTCTGCCGTTAAAGAATGGAGC
TTAACTCCATAGGAGCGGTGGAAACTGCAAGACTAGAGTACAGTAGGGGTAGCAGGAATTCCCAGTGTAGCGGTGAAATGCGTAGATATTGGGA
AGAACATCGGTGGCGAAAGCGTGCTACTGGGCTGAAACTGACACTGAGGGACGAAAGCTAGGGTAGCGAAAGGGATTAGATACCCCTGTAGTCC
TAGCCGTAAACGATGGATACTAGGCGTGGCTTGTATCGACCCGAGCCGTGCCGAAGCTAACGCGTTAAGTATCCCGCCTGGGGAGTACGCACGC
AAGTGTGAAACTCAAAGGAATTGACGGGGGCCCGCACAAGCGGTGGAGTATGTGGTTTAATTCGATGCAACGCGAAGAACCTTACCAAGGCTTG
ACATCCCTGGAATCCTGCGGAAACGTGGGAGTGCCTTAGGGAGCCAGGAGACAGGTGGTGCATGGCTGTCGTCAGCTCGTGTCGTGAGATGTTG
GGTTAAGTCCCGCAACGAGCGCAACCCTCGTTTTTAGTTGCCATCATTAAGTTGGGCACTCTAGAGAGACTGCCGGTGACAAACCGGAGGAAGG
TGGGGATGACGTCAAGTCATCATGCCCCTTACGCCTTGGGCTACACACGTACTACAATGGTCGGGACAACGGGCAGCGAGCTCGCGAGAGTAAG
CGAATCCCATCAAACCCGGCCTCAGTTCAGATTGCAGGCTGCAACTCGCCTGCATGAAGGAGGAATCGCTAGTAATCGCAGGTCAGAATACTGC
GGTGAATTCGTTCCCGGGCCTTGTACACACCGCCCGTCACACCATGGGAGCTGGTCACGCCCGAAGTCGTTACTCTAACCTTAGGGAGGAGGGC
GCCGAAGGCAGGGCTAGTGACTGGGGTGAAGTCGTAACAAGGTAGCCGTACCGGAAGGTGTGGCTGGATCACCTCCTTT

>ENA|CP000100.1:2658843..2660332:rRNA|CP000100.1:2658843..2660332:rRNA.1 Synechococcus elongatus PCC 7942 16S ribosomal RNA
CAAAATGGAGAGTTTGATCCTGGCTCAGGATGAACGCTGGCGGCGTGCTTAACACATGCAAGTCGAACGGGCTCTTCGGAGCTAGTGGCGGACG
GGTGAGTAACGCGTGAGAATCTGCCTACAGGACGGGGACAACAGTTGGAAACGACTGCTAATACCCGATGTGCCGAGAGGTGAAACATTTATGG
CCTGTAGATGAGCTCGCGTCTGATTAGCTAGTTGGTGGGGTAAGGGCCTACCAAGGCGACGATCAGTAGCTGGTCTGAGAGGATGATCAGCCAC
ACTGGGACTGAGACACGGCCCAGACTCCTACGGGAGGCAGCAGTGGGGAATTTTCCGCAATGGGCGCAAGCCTGACGGAGCAACGCCGCGTGGG
GGAGGAAGGTTTTTGGACTGTAAACCCCTTTTCTCAGGGAAGAAGAAAGTGACGGTACCTGAGGAATAAGCCTCGGCTAATTCCGTGCCAGCAG
CCGCGGTAATACGGGAGAGGCAAGCGTTATCCGGAATTATTGGGCGTAAAGCGTCCGCAGGCGGTTAATCAAGTCTGTTGTCAAAGCGTGGGGC
TCAACCTCATACAGGCAATGGAAACTGATTGACTAGAGTATGGTAGGGGTAGCGGGAATTCCAGGTGTAGCGGTGAAATGCGTAGATATCTGGA
AGAACACCAGCGGCGAAAGCGCGCTACTGGGCCATAACTGACGCTCATGGACGAAAGCTAGGGGAGCGAAAGGGATTAGATACCCCTGTAGTCC
TAGCCGTAAACGATGAACACTAGGTGTTGCGTGAATCGACCCGCGCAGTGCCGTAGCCAACGCGTTAAGTGTTCCGCCTGGGGAGTACGCACGC
AAGTGTGAAACTCAAAGGAATTGACGGGGGCCCGCACAAGCGGTGGAGTATGTGGTTTAATTCGATGCAACGCGAAGAACCTTACCAGGGTTTG
ACATCCCCCGAATCTCTTGGAAACGAGAGAGTGCCTTCGGGAGCGGGGAGACAGGTGGTGCATGGCTGTCGTCAGCTCGTGTCGTGAGATGTTG
GGTTAAGTCCCGCAACGAGCGCAACCCACGTTTTTAGTTGCCATCATTCAGTTGGGCACTCTAGAGAAACTGCCGGTGACAAACCGGAGGAAGG
TGTGGACGACGTCAAGTCATCATGCCCCTTACATCCTGGGCTACACACGTACTACAATGCTCCGGACAGCGAGACGCGAAGCCGCGAGGTGAAG
CAAATCTCCCAAACCGGGGCTCAGTTCAGATTGCAGGCTGCAACTCGCCTGCATGAAGGCGGAATCGCTAGTAATCGCAGGTCAGCATACTGCG
GTGAATACGTTCCCGGGCCTTGTACACACCGCCCGTCACACCATGGAAGTTGGCCATGCCCGAAGTCGTTACCCTAACCGTTCGCGGAGGGGGG
CGCCGAAGGTAGGGCTGATGACTGGGGTGAAGTCGTAACAAGGTAGCCGTACCGGAAGGTGTGGCTGGATCACCTCCTTT

>ENA|BA000019.2:2500525..2502013:rRNA|BA000019.2:2500525..2502013:rRNA.2 Nostoc sp. PCC 7120 = FACHB-418 16S rRNA
AAAACGGAGAGTTTGATCCTGGCTCAGGATGAACGCTGGCGGTATGCTTAACACATGCAAGTCGAACGGTCTCTTCGGAGATAGTGGCGGACGG
GTGAGTAACGCGTGAGAATCTAGCTTCAGGTCGGGGACAACCACTGGAAACGGTGGCTAATACCGGATGTGCCGAAAGGTGAAAGATTTATTGC
CTGAAGATGAGCTCGCGTCTGATTAGCTAGTTGGTGTGGTAAGAGCGCACCAAGGCGACGATCAGTAGCTGGTCTGAGAGGATGATCAGCCACA
CTGGGACTGAGACACGGCCCAGACTCCTACGGGAGGCAGCAGTGGGGAATTTTCCGCAATGGGCGAAAGCCTGACGGAGCAATACCGCGTGAGG
GAGGAAGGCTCTTGGGTTGTAAACCTCTTTTCTCAGGGAATAAAAAAATGAAGGTACCTGAGGAATAAGCATCGGCTAACTCCGTGCCAGCAGC
CGCGGTAATACGGAGGATGCAAGCGTTATCCGGAATGATTGGGCGTAAAGCGTCCGCAGGTGGCACTGTAAGTCTGCTGTTAAAGAGCAAGGCT
CAACCTTGTAAAGGCAGTGGAAACTACAGAGCTAGAGTACGTTCGGGGCAGAGGGAATTCCTGGTGTAGCGGTGAAATGCGTAGAGATCAGGAA
GAACACCGGTGGCGAAAGCGCTCTGCTAGGCCGTAACTGACACTGAGGGACGAAAGCTAGGGGAGCGAATGGGATTAGATACCCCAGTAGTCCT
AGCCGTAAACGATGGATACTAGGCGTGGCTTGTATCGACCCGAGCCGTGCCGGAGCCAACGCGTTAAGTATCCCGCCTGGGGAGTACGCACGCA
AGTGTGAAACTCAAAGGAATTGACGGGGGCCCGCACAAGCGGTGGAGTATGTGGTTTAATTCGATGCAACGCGAAGAACCTTACCAAGACTTGA
CATGTCGCGAATCTTCTTGAAAGGGAAGAGTGCCTTAGGGAGCGCGAACACAGGTGGTGCATGGCTGTCGTCAGCTCGTGTCGTGAGATGTTGG
GTTAAGTCCCGCAACGAGCGCAACCCTCGTTTTTAGTTGCCAGCATTAAGTTGGGCACTCTAGAGAGACTGCCGGTGACAAACCGGAGGAAGGT
GGGGATGACGTCAAGTCAGCATGCCCCTTACGTCTTGGGCTACACACGTACTACAATGCTACGGACAGAGGGCAGCAAGCTAGCGATAGCAAGC
AAATCCCGTAAACCGTAGCTCAGTTCAGATCGCAGGCTGCAACTCGCCTGCGTGAAGGAGGAATCGCTAGTAATTGCAGGTCAGCATACTGCAG
TGAATTCGTTCCCGGGCCTTGTACACACCGCCCGTCACACCATGGAAGCTGGCAACGCCCGAAGTCATTACTCCAACTTTTAGGAGAGGAGGAT
GCCTAAGGCAGTGCTGGTGACTGGGGTGAAGTCGTAACAAGGTAGCCGTACCGGAAGGTGTGGCTGGATCACCTCCTTT

>ENA|CP003591.1:235886..237361:rRNA|CP003591.1:235886..237361:rRNA.1 Geitlerinema sp. PCC 7407 16S ribosomal RNA
AGAGTTTGATCCTGGCTCAGGATGAACGCTGGCGGCGTGCTTAACACATGCAAGTCGAACGAAGTCTTCGGACTTAGTGGCGGACGGGTGAGTA
ACGCGTGAGAATCTGCCCTCAGGAGGGGGATAACAGTTGGAAACGACTGCTAAGACCCCATATGCCGAAAGGTGAAATAATTTTTTGCCTGAGG
ATGAGCTCGCGTCTGATTAGCTAGTTGGTGAGGTAAGAGCTCACCAAGGCGACGATCAGTAGCTGGTCTGAGAGGATGATCAGCCACACTGGGA
CTGAGACACGGCCCAGACTCCTACGGGAGGCAGCAGTGGGGAATTTTCCGCAATGGGCGAAAGCCTGACGGAGCAACGCCGCGTGAGGGAGGAA
GGCCTGTGGGTTGTAAACCTCTTTTCTCAGGGAAGAAGATCTGACGGTACCTGAGGAATCAGCATCGGCTAACTCCGTGCCAGCAGCCGCGGTA
AGACGGAGGATGCAAGCGTTATCCGGAATTATTGGGCGTAAAGCGTCCGCAGGCGGCTCAGCAAGTCTGCTGTCAAAGAGCGGGGCTCAACTCC
GTAGGGGCAGTGGAAACTGCTGGGCTAGAGTATGGTAGGGGCAGAGGGAATTCCCGGTGTAGCGGTGAAATGCGTAGATATCGGGAAGAACACC
AGTGGCGAAAGCGCTCTGCTGGGCCATAACTGACGCTCATGGACGAAAGCTAGGGGAGCGAATGGGATTAGATACCCCAGTAGTCCTAGCCGTA
AACGATGGAGACTAGGTGTTGCTTGTATCGACCCAAGCAGTGCCGTAGCTAACGCGTTAAGTCTCCCGCCTGGGGAGTACGCACGCAAGTGTGA
AACTCAAAGGAATTGACGGGGGCCCGCACAAGCGGTGGAGTATGTGGTTTAATTCGATGCAACGCGAAGAACCTTACCAAGGCTTGACATGTCG
CGAACCCTGCTGAAAGGCGGGGGTGCCTACGGGAGCGCGAACACAGGTGGTGCATGGCTGTCGTCAGCTCGTGTCGTGAGATGTTGGGTTAAGT
CCCGCAACGAGCGCAACCCTCGTTCTTAGTTGCCATCATTAAGTTGGGCACTCTAGGGAGACTGCCGGTGACAAACCGGAGGAAGTGGGGATGA
CGTCAAGTCAGCATGCCCCTTACGTCTTGGGCTACACACGTACTACAATGCTTCGGACAGAGGGCAGCAAGCGCGCGAGTGCAAGCCAATCCCA
TAAACCGAGGCTCAGTTCAGATTGCAGGCTGCAACTCGCCTGCATGAAGGCGGAATCGCTAGTAATCGCAGGTCAGCATACTGCGGTGAATACG
TTCCCGGGCCTTGTACACACCGCCCGTCACACCATGGGAGCTGGCCACGCCCGAAGTCGTTACTCTAACCGTTCGCGGAGGAGGACGCCGAAGG
CAGGGCTGGTGACTGGGGTGAAGTCGTAACAAGGTAGCCGTACCGGAAGGTGTGGCTGGATCACCT

>2663544969 16S rRNA. Bacterial SSU [Oscillatoria limnetica bin re-assembly (V2) : Ga0115370_1063] (+)strand
ACACCACGGAGAGTTTGATCCTGGCTCAGGATGAACGCTGGCGGTCGGCTTAACACATGCAAGTCGAACGGGTTCTTCGGAACCAGTGGCGGAC
GGGTGAGTAACACGTGAGAACCTGCCTACCGGAGGGGGATAACAGCGGGAAACTGCTGCTAATACCCCATACGCCGAGAGGTAAAAGGTCAGCC
GCCGGGAGAGGGGCTCGCGTCCGATTAGCTAGTTGGTGGGGTAAGAGCTCACCAAGGCGACGATCGGTAGCCGTTTTGAGAGGAAGAACGGCCA
CACTGGGACTGAGACACGGCCCAGACTCCTACGGGAGGCAGCAGTGGGGAATCTTCCGCAATGGGCGCAAGCCTGACGGAGCCAGACCGCGTGG
GGGAGGAAGGCCCTTGGGTTGTAAACCCCTTTTCTCAGGGAAGAAACCTGACGGTACCTGAGGAAAAAGCCTCGGCTAACTCCGTGCCAGCAGC
CGCGGTAATACGGAGGAGGCAAGCGTTATCCGGAATTACTGGGCGTAAAGCGTTCGTAGGGGGCTCGTCAAGTCTGCTGTCAAAGCTCGGAGCT

CAACTCCGAAGGGGCAGTGGAAACTGGGGAGCTAGAGGGCGGCAGGGGCAGAAGGAATTCCCGGTGTAGCGGTGAAATGCGTAGAGATCGGGAA
GAACACCAGAGGCGAAAGCGTTCTGCTGGGCCGCGCCTGACCCTGAGGGACGAAAGCCAGGGGAGCGAATGGGATTAGATACCCCAGTAGTCCT
GGCTGTAAACGATGGATACTAGGTATTGGCCGTATCGACCCGGTCGGCGCCGAAGCTAACGCGTTAAGTATCCCGCCTGGGGAGTACGCACGCA
AGTGTGAAACTCAAAGGAATTGACGGGGGCCCGCACAAGCGGTGGAGTATGTGGTTTAATTCGATGCAACGCGAAGCACCTTACCAGGGCTTGA
CATGCCGCGAACCCCAGGGAAACTTGGGGGTGCCTACGGGAGCGCGGACACAGGTGGTGCATGGCTGTCGTCAGCTCGTGTCGTGAGATGTTGG
GTTAAGTCCCGCAACGAGCGCAACCCTCGTCCTTAGTTGCCAGCATTCAGTTGGGCCCTCTAGGGAGACTGCCGGTGACAAACCGGAGGAAGGT
GGGGATGACGTCAAGTCATCATGCCCCTTATGCCCTGGGCGACACACGTACTACAATGGTTGGGACAGAGGGAAGCGAAGCCGCGAGGTGGAGC
CAATCCCACAAACCCAGCCTCAGTTCAGATTGCAGGCTGCAACTCGCCTGCATGAAGGCGGAATCGCTAGTAATCGCCGGTCAGCATACGGCGG
TGAATACGTTCCCGGGCCTTGTACACACCGCCCGTCACACCATGGAAGCTGGCCACGCCCGAAGTCGTTACTCCAACCCTGAGGGGAGGAGGAC
GCCGAAGGCAGGGCTGGTGACTGGGGTGAAGTCGTAACAAGGTAGCCGTACCGGAAGGTGTGGCTGGATCACCTCCTTTA

> Geitlerinema sp. ZHR1A, 16S rRNA (fragment) obtained using CYA 359F and CYA 781R
TGTGGGAATTTTCCGCAATGGGCGAAAGCCTGACGGAGCAATACCGCGTGAGGGACGAAGGCCTGTGGGTTGTAAACCTCTTTTCT
CAGGGAAGAAGATCTGACGGTACCTGAGGAATCAGCATCGGCTAACTCCGTGCCAGCAGCCGCGGTAAGACGGAGGATGCAAGCGT
TATCCGGAATTATTGGGCGTAAAGCGTCCGCAGGCGGTCTCGTAAGTCTGTCTTTAAAGCGTGGAGCTTAACTCCATAAAGGGGAT
GGAAACTGCGAGACTAGAGGTAGGTAGGGGTAGAAGGAATTCCCAGTGTAGCGGTGAAATGCGTAGATATTGGGAAGAACACCAGC
AGCGAAGGCGTTCTACTGGACCAAACCTGACGCTCATGGACGAAAGCTAGGGGAGCGAAWGGGATTAGATACCCCWGTAGTCAA

>ENA|HE974210.1:257..1733:rRNA|HE974210.1:257..1733:rRNA.1 Microcystis aeruginosa PCC 9701 ribosomal RNA 16s ribosomal RNA
AGAGTTTGATCCTGGCTCAGGATGAACGCTGGCGGCGTGCCTAACACATGCAAGTCGAACGGGAATCTTCGGATTCTAGTGGCGGACGGGTGAG
TAACGCGTAAGAATCTAACTTCAGGACGGGGACAACAGTTGGAAACGACTGCTAATACCCGATGTGCCGCAAGGTGAAACCTAATTGGCCTGAA
GAAGAGCTTGCGTCTGATTAGCTAGTTGGTGGGGTAAAGGCCTACCAAGGCGACGATCAGTAGCTGGTCTGAGAGGATGAGCAGCCACACTGGG
ACTGAGACACGGCCCAGACTCCTACGGGAGGCAGCAGTGGGGAATTTTCCGCAATGGGCGAAAGCCTGACGGAGCAACGCCGCGTGAGGGAGGA
AGGTCTTTGGATTGTAAACCTCTTTTCTCAAGGAAGAAGTTCTGACGGTACTTGAGGAATCAGCCTCGGCTAACTCCGTGCCAGCAGCCGCGGT
AATACGGGGGAGGCAAGCGTTATCCGGAATTATTGGGCGTAAAGCGTCCGCAGGTGGTCAGCCAAGTCTGCTGTCAAATCAGGTTGCTTAACGA
CCTAAAGGCGGTGGAAACTGGCAGACTAGAGAGCAGTAGGGGTAGCAGGAATTCCCAGTGTAGCGGTGAAATGCGTAGAGATTGGGAAGAACAT
CGGTGGCGAAAGCGTGCTACTGGGCTGTATCTGACACTCAGGGACGAAAGCTAGGGGAGCGAAAGGGATTAGATACCCCTGTAGTCCTAGCCGT
AAACGATGGATACTAGGCGTGGCTTGTATCGACCCGAGCCGTGCCGAAGCTAACGCGTTAAGTATCCCGCCTGGGGAGTACGCACGCAAGTGTG
AAACTCAAAGGAATTGACGGGGGCCCGCACAAGCGGTGGAGTATGTGGTTTAATTCGATGCAACGCGAAGAACCTTACCAAGACTTGACATGTC
GCGAACCCTGGTGAAAGCTGGGGGTGCCTTCGGGAGCGCGAACACAGGTGGTGCATGGCTGTCGTCAGCTCGTGTCGTGAGATGTTGGGTTAAG
TCCCGCAACGAGCGCAACCCTCGTTCTTAGTTGCCAGCATTAAGTTGGGGACTCTAAGGAGACTGCCGGTGACAAACCGGAGGAAGGTGGGGAT
GACGTCAAGTCAGCATGCCCCTTACGTCTTGGGCGACACACGTACTACAATGGTCGGGACAAAGGGCAGCGAACTCGCGAGAGCCAGCGAATCC
CAGCAAACCCGGCCTCAGTTCAGATTGCAGGCTGCAACTCGCCTGCATGAAGGAGGAATCGCTAGTAATCGCCGGTCAGCATACGGCGGTGAAT
TCGTTCCCGGGCCTTGTACACACCGCCCGTCACACCATGGAAGCTGGTCACGCCCGAAGTCATTACCTCAACCGCAAGGAGGGGGATGCCTAAG
GCAGGGCTAGTGACTGGGGTGAAGTCGTAACAAGGTAGCCGTACCGGAAGGTGTGGCTGGATCACCT

>ENA|CP003607.1:2196585..2197955:rRNA|CP003607.1:2196585..2197955:rRNA.1 Oscillatoria acuminata PCC 6304 16S ribosomal RNA
CTGGCTCAGGATGAACGCTGGCGGTCTGCTTAACACATGCAAGTCGAACGGTGAACCCTCGGGTTCATAGTGGCGGACGGGTGAGTAACACGTG
AGAATCTGCCTCCAGGTCGGGGACAACAGTGGGAAACTGCTGCTAATACCCGATGTGCCTACGGGTGAAAGATTAATTGCCTGGAGATGAGCTC
GCGTCCGATTAGCTAGTTGGTAGAGTAAAAGCCTACCAAGGCGACGATCGGTAGCTGGTCTGAGAGGATGAGCAGCCACACTGGGACTGAGACA
CGGCCCAGACTCCTACGGGAGGCAGCAGTGGGGAATTTTCCGCAATGGGCGAAAGCCTGACGGAGCAAGACCGCGTGGGGGATGAAGGCTCTTG
GGTTGTAAACCCCTTTTCTCAGGGAAGAATAACTGACGGTACCTGAGGAATAAGCATCGGCTAACTCCGTGCCAGCAGCCGCGGTAAGACGGAG
GATGCAAGCGTTATCCGGAATTATTGGGCGTAAAGCGTTCGTAGGTGGCTGTTCAAGTCTGCCGTTAAAGACCGGGGCTTAACTCCGGAAAAAC
TGTGGAAACTGAACAGCTAGAGTATGGTAGGGGTAAGGGGAATTCCCGGTGTAGCGGTGAAATGCGTAGATATCGGGAAGAACACCGGTGGCGA
AGGCGCCTTACTGGGCCATAACTGACACTGAGGGACGAAAGCTAGGGGAGCGAAAGGGATTAGATACCCCTGTAGTCCTAGCTGTAAACGATGG
ATACTAGGTGTTGCCCGTATCGACCCGGGCAGTGCCGTAGCTAACGCGTTAAGTATCCCGCCTGGGGAGTACGCACGCAAGTGTGAAACTCAAA
GGAATTGACGGGGGCCCGCACAAGCGGTGGAGTATGTGGTTTAATTCGATGCAACGCGAAGAACCTTACCAGGGCTTGACATGTCCAGAATCTT
GGGGAAACCTAAGAGTGCCTTCGGGAGCTGGAACACAGGTGGTGCATGGCTGTCGTCAGCTCGTGTCGTGAGATGTTGGGTTAAGTCCCGCAAC
GAGCGCAACCCTCGTCCTTAGTTGCCATCATTAAGTTGGGCACTTTAGGGAGACTGCCGGTGACAAACCGGAGGAAGGTGGGGATGACGTCAAG
TCAGCATGCCCCTTACGTCCTGGGCTACACACGTACTACAATGGGAAGGACAGAGGGTAGCAAGCGCGCGAGTGCAAGCCAATCCCATAAACCT
TTCCTCAGTTCAGATTGAAGTTTGCAACTCAACTTCATGAAGGCGGAATCGCTAGTAATCGCAGGTCAGCATACTGCGGTGAATACGTTCCCGG
GCCTTGTACACACCGCCCGTCACACCATGGAAGTTGGCCATGCCCGAAGTCATTA

>J01395.1:223-1714 Chlamydomonas reinhardtii 16S ribosomal RNA gene, complete sequence;
ATCCATGGAGAGTTTGATCCTGGCTCAGGACGAACGCTGGCGGCATGCTTAACACATGCAAGTCGAACGAGCAAAGCAATTTGTGTAGTGGCGA
ACGGGTGCGTAACGCGTAAGAACCTACCTATCGGAGGGGGATAACATTGGGAAACTGTTGCTAATACCCCATACAGCTGAGGAGTGAAAGGTGA
AAAACCGCCNGATAGAGGGGCTTGCGTCTGATTAGCTAGTTGGTGGGGGTAACGGCCTCCCAAGGCCACGAGCAGTAGCTGGTCTGAGAGGATG
ATCAGCCACACTGGGACTGAGACACGGCCCAGACTCCTACGGGAGGCAGCAGTGAGGAATTTTTCGCAATGGGCGCAAGCGACGGAGCAATGCC
GCGTGCAGGAAGAAGGCCTGTGGGTCGTAAACTGCTTTTCTCAGAGAAGAAGTTCTGACGGTATCTGAGGAATAAGCACCGGCTAACTCTGTGC
CAGCAGCCGCGGTAATACAGAGGGTGCAAGCGTTGTCCGCAATGATTGGGCGTAAAGCGTCTGTAGGTGGCTCGTAAAGTCTAATGTCAAATAC
CAGGGCTCAACCTTGGACCGGCATTGGAGTACTCACGAGCTTGAGTACGGTAGGGGCAGAGGGAATTCCATGTGGAGCGGTGAAATGCGTAGAG
ATATGGAGGAACACCAGTGGCGAAGGCGCTCTGCTGGGCCGAAACTGACACTGAGAGACGAAAGCTGGGGGAGCGAATAGGATTAGATACCCTA
GTAGTCCCAGCCGTAAACTATGGAGACTAAGTGCTGCCGCAAGCAGTGCTGTAGCTAACGCGTTAAGTCTCCCGCCTGGGGAGTATGCTCGCAA
GAGTGAAACTCAAAGGAATTGACGGGACCGCACAAGCGGTGGATTATGTGGATTAATTCGATACAACGCGAAGAACCTTACCAGGGTTTGACAT
GTCAAGAANCCTCTCAGAAATGGGAGGGTGCCCTAACGGACTTGAACACAGGTGGTGCATGGCTGTCGTCAGCTCGTGCTGTGAAGTGTATAGT
TAAGTCTCATAACGAGCGCAACCCTCGTCTTTAGTTGCCATTNNNNNNNNTGGTTCTCTAAAGAGACTGCCAGTGTNAAGCTGGAGGAAGGTGA
GGATGACGTCAAGTCAGCATGCCCCTTACATCCTGGGCTTCACACGTAATACAATGGTTGGGACAATCAGAAGCGANCTCGTGAGNNNNAGCTA
GCGGCTCTGTTAAACCCAACCTCAGTTCGGATTGTAGGCTGCAACTCGCCTACATGAAGCCGGAATCGCTAGTAATCGCCAGTCAGCTATATGG
CGGTGAATACGTTCCCGGGTCTTGTACACACCGCCCGTCACACCATGGAAGCTGGTTCTGCTCCAAGTCGTTACCCTAACCTTCGGGAGGGGGG
CNNGCCTAAAGCAGGGCTAGTGACTAGGGTGAAGTCGTAACAAGGTAGGGCTACTGGAAGGTGGCCCTGGCTCACCTCCTTC

>ENA|GU084820.1:229350..230840:rRNA|GU084820.1:229350..230840:rRNA.1 Volvox carteri f. nagariensis 16S ribosomal RNA
ATCCATGGAGAGTTTGATCCTGGCTCAGGACGAACGCTGGCGGCATGCTTAACACATGCAAGTCGAACGAGCATTAAATTTTTAATGTGTAGTG
GCGAACGGGTGCGTAACGCGTAAGAACCTACCTATCGGAGGGGGATAACATTGGGAAACTGTTGCTAATACCCCATAAAAGCTGAGGAGTGAAA

GGTGTAAAACCGCCGATAGAGGGGCTTGCGTCTGATTAGCTAGTTGGAGGGGGTAATGGCCTCCCAAGGCCACGATCAGTAGCTGGTCTGAGAG
GATGATCAGCCACACTGGGACTGAGACACGGCCCAGACTCCTACGGGAGGCAGCAGTGAGGAATTTTTCGCAATGGGCGAAAGCCTGACGAAGC
AATGCCGCGTGCAGGAAGAAGGCCTGTGGGTCGTAAACTGCTTTTCTTAGAGAAGAAGTTCTGACGGTATCTGAGGAATAAGCACCGGCTAACC
CTGTGCCAGCAGCCGCGGTAATACAGGGGGTGCAAGCGTTGTCCGGAATGATTGGGCGTAAAGCGTCTGTAGGTGGCTCGTAAAGTCTAATGTC
AAATACCAGGGCTCAACCTTGGATCGGCATTAGAGCACTCACGAGCTTGAGTACGGTAGAGGCAGAGGGAACTCCAAGTGGAGCGGTGAAATGC
GTAGAGATTTGGAAGAACACCAGAGGCGAAGGCGCTCTGCTGGGCCGAAACTGACACTGAGAGACGAAAGCTGGGGGAGCGAATAGGATTAGAT
ACCCTAGTAGTCCCAGCCGTAAACTATGGAGACTAAGTGCTGCCGCAAGCAGTGCTGTAGCTAACGCGTTAAGTCTCCCGCCTGGGGAGTATGC
TCGCAAGAGTGAAACTCAAAGGAATTGACGGGGACCCGCACAAGAGGTGGATTATGTGGATTAATTCGATGCAACGCGAAGAACCTTACCAGGG
TTTGACATGTCAAGAACTTCCCAGAAATGGAGGGTGCCCTAACGGGAGCTTGAACACAGGTGGTGCATGGCTGTCGTCAGCTCGTGCCGTGAG
GTGTATAGTTAAGTCTCATAACGAGCGCAACCCTCGTCTTTAGTTGCCATTTGGTTCTCTAAAGAGACTGCCAGTGTAAGCTGGAGGAAGGTGA
GGATGACGTCAAGTCAGCATGCCCCTTACACCCTGGGCTTCACACGTAATACAATGGTTGGGACAATCAGAAGCGATCTCGTGAGAGCTAGCGG
ATCTGTTAAACCCAACCTCAGTTCGGATTGTAGGCTGCAACTCGCCTACATGAAGCCGGAATCTCTAGTAATCGCCAGTCAGCTATATGGCGGT
GAATACGTTCCCGGGTCTTGTACACACCGCCCGTCACAGGCCGAAAATTAACTCAACTTTAAGCAGTGAGCTGAACCTGCAAAGCAGGACGGGA
GCTACCACAGTGGGGTTAGTAATTGATCTGAAGTCGTAACAAGGTAGGGCTACTGGAAGGTGGCCCTGGATCACCTCCTTC

>ENA|AF170718|AF170718.1 Galdieria sulphuraria strain SAG 107.79 culture-collection SAG:107.79
16S small subunit ribosomal RNA gene, partial sequence; plastid.
TGTGCTGCAGAGAGTTTGATCCTGGCTCAGGATTAACGCTGGCGGTATGCCTAACACATGCAAGTCGTACGAGAATTTTATTCTAGTGGCGGAC
GGGTGAGTAACACGTGAGAATCTACCTCTAGGAGGGGGATAACAGTTGGAAACGATTGCTAAAACCCCATATGCCATTATTGGTGAAAAAGATT
TATCTGCCTGGAGATGAGCTCGCGGCTGATTAGCTAGTTGGTAGGGTAATGGCTTACCAAGGCAACGATCAGTAGCTGGTCTTAGAGGATGATC
AGCCACACTGGAACTGAGATATGGTCCAGACTCCAACGGGAGGCAGCAGAGGGGAATTTTCCACAATGGGCGAAAGCCTGATGGAGCAATACCG
TGTGAGGGATGAAGGCCTGTGGGTTGTAAACCTCTTTTTTCAGGAAAGAAACTTTGACGGTACCTGAAGAATAAGCATCGGCTAACTCCGTGCC
AGCAGCCGCGGTAATACGGAGGATGCAAGCGTTATCCGGAATCACTGGGCGTAAAGCGTCTGTAGGTGGTTTATCAAGTCTGCTGTTAAAGCTT
GAGGCTTAACCTCAAAAAAGCAGTGGAAACTGATAGACTAGAGAATGGTAGGGGCAGAGAGAATTCTCAGTGTAGCGGTGAAATGCGTAGATAT
TGAGAAGAATACCGATAGCGAAGGCGCTCTGCTGGGCCATTACTGACACTCAGAGACGAAAGTTAGGGGAGCAAATGGGATTAGATACCCCAGT
AGTCCTAGCCGTAAACTATGGATACTAGATGTTGTGTGAGTAAAATTGTGCAGTATCGAAGCTAACGCGTTAAGTATCCCGCCTGGGAAGTACG
CTCGCAAGAGTGAAACTCAAAGGAATTGACGGGGACCCGCACAAGCGGTGGAGCATGTGGTTTAATTCGATGCAACGCGAAGAACCTTACCAGG
ACTTGACATGTTACTAATTTCCTTGAAAGAGGAAAGTGCCTTTGGGAAAGTAAACACAGGTGGTGCATGGCTGTCGTCAGCTCGTGTCGTGAGA
TGTTGGGTTAAGTCCCGCAACGAGCGCAACCCTTGTCTTTAGTTACCATCATTAAGTTGGGGACTCTAAAGAGACTGCCGGTGATAAACCGGAG
GAAGGTAAGGATGAGGTCAAGTCATCATGCCCCTTATGTCCTGGGCTACACACGTGCTACAATGGTTAGGACAATAAGTCGCAAATTCGTGAGA
ACTAGCTAATCTTATAAACCTAATCTCAGTACGGATTGTAGGCTGCAACTCGCCTACATGAAGACGGAATCGCTAGTAATCGCTGGTCAGCTAC
ACAGCGGTGAATACGTTCCCGGGTCTTGTACACACCGCCCGTCACACCATGGGAGCTGGCCATGTCCGAAATCATTACTCTAACCTTAATGGAG
GAGGATGCTTAAGGCAGGGCTAGTGACTGGGG

>ENA|AP012987.1:208682..210168:rRNA|AP012987.1:208682..210168:rRNA.1 Porphyridium purpureum
16S small subunit ribosomal RNA
ATACCATGGAGAGTTTGATCCTGGCTCAGGATGAACGCTGGCGGTATGCTTAACACATGCAAGTCGTACGAAAACTTCGGTTTTAGTGGCGGAC
GGGTGAGTAACACGTGAGAATCTGCCTTTAGGAGGGGGATAACAACTAGAAATGGTTGCTAATACCCCATATGCCGCAAGGTGAAACAGTTTTC
TGCCTATAGAAGAGCTCGCGTCTGATTAGCTTGTTGGTGAGGTAATGGCTCACCAAGGCGACGATCAGTAGTTGGTCTGAGAGGATGGCCAGCC
ACACTGGGACTGAGACACGGCCCAGACTCCTACGGGAGGCAGCAGTGGGGAATTTTCCGCAATGGGCGAAAGCCTGACGGAGCAATACCGCGTG
AGGGATGAAGGCTCGTGGGTTGTAAACCTCTTTTCTCAGGAAAGAAGTTCTGACGGTACCTGAGGAATAAGCATCGGCTAACTCCGTGCCAGCA
GCCGCGGTAATACGGGGGATGCAAGCGTTATCCGGAATCACTGGGCGTAAAGCGTCTGTAGGTTGTTCAGTAAGTCTAATGTTAAAGACTAGGG
CTCAACCCTGGAAAAGCATTAGAAACTACTAGACTAGAGTATGGTAGAGGTAGAGGGAATTCCCAGTGTAGCGGTGAAATGCGTAGATATTGGG
AAGAACACCAGTGGCGAAGGCGCTCTACTGGGCCATAACTGACACTGAGAGACGAAAGCTAAGGGAGCGAATAGGATTAGATACCCTAGTAGTC
TTAGCTGTAAACGATGGATACTAGATGTTGCGTGTTTTTTCATGCAGTGTCGTAGCTAACGCGTTAAGTATCCCGCCTGGGGAGTATGCTCGCA
AGGGTGAAACTCAAAGGAATTGACGGGGGCCCGCACAAGCGGTGGAGCATGTGGTTTAATTTGATGCAACGCGGAGAACCTTACCAGAACTTGA
CATATCATGAATCTTTATTAATGTAAAGAGTGTCTTCGGAAACATGAATACAGGTGGTGCATGGCTGTCGTCAGCTCGTGTCGTGAGATGTTGG
GTTAAGTCCCGCAACGAGCGCAACCCTTGTCTTTAGTTGCCATCATTAAGTTGGGGACTCTAAAGAGACTGCCGGTGACAAACCGGAGGAAGGT
GGGGATGACGTCAAGTCAGCATGCCCCTTACGTTCTGGGCTACACACGTGCTACAATGGTTAGAACAAAGAGTTGCAAACTTGTGAAAGTAAGC
TAATCTCATAAATCTAGCCTCAGTTCAGATTGTAGGCTGAAACTCGCCTACATGAAGGTGGAATCGCTAGTAATCGCCGGTCAGCTACACGGCG
GTGAATTCGTTCCCGGGCCTTGTACACACCGCCCGTCACACCATGGAAGCTGGCTATGCCCAAAGTCGTTACTCTAACCGTAAGGAGGGGGGCG
CCTAAGGCAGAGCTAGTGACTGGGGTGAAGTCGTAACAAGGTAGCCGTACTGGAAGGTGCGGCTGGATCACCTCCTT

>X12742.1 Chlorella ellipsoidea chloroplast 16S rDNA
TTTCATGGAGAGTTTCGATCCTGGCTCAGGATGAACGCTGGCGGCATGCTTAACACATGCAAGTCGAACGGAGGTTTGCTTTTTTTAAGCTTAG
GTTAAAAAAAAGTAAATTCTTAGTGGCGGACGGGTGAGTAACGCGTAAGAACCTACCTTTAGGCGGGGGACAACAACTGGAAACGGTTGCTAAT
ACCCCATAAGCTGAGGAGTAAAAGGAATACTATTCTGTCTAAAGATGGGCTTGCGTCTGATTAGCTTGTTGGTGAGGTAATTGCTTACCAAGGC
AACGATCAGTAGCTGGTCTGAGAGGATGATCAGCCACACTGGGACTGAGACACGGCCCAGACTTCTACGGAAGGCAGCAGTGAGGAATTTTCCG
CAATGGGCGAAAGCTGACGGAGCAATGCCGCGTGGAGGATGACAGCCTGTGGGTCGTAAACTCCTTTTCTCAGAGAAGAAGTTCTGACGGTATC
TGAGGAATAAGCATCGGCTAACTCCGTGCCAGCAGCCGCGGTAAGACGGAGGATGCAAGCGTTATCCGGAATGATTGGGCGTAAAGCGTCTGTA
GGTGGTTTATTAAGTCTACTGTTAAAGATCAGGGCTTAACCCTGAGTCGGCAGTAGAAACTAATGAGCTTGAGTACGGTAGGGGCAGAGGGAAT
TCCGGGTGTAGCGGTGAAATGCGTAGAGATCGGGAAGAACACCAATGGCGAAAGACTCTGCTGGGCCGAAACTGACACTCAGAGACGAAAGCTA
GGGGAGCGAATGGGATTAGATACCCCAGTAGTCCTAGCCGTAAACGATGGATACTAAGTGCTGTGCGACTCAAAACGTCCAGTACTGTAGCTAA
CGCGTGAAGTATCCCGCCTGGGGAGTATGCTCGCAAGAGTGAAACTCAAAGGAATTGACGGGGGCCCGCACAAGCGGTGGAGCATGTGGTTTAA
TTCGATGCAACGCGAAGAACCTTACCAGGGCTTGACATGCCATTTAATCTCTTGAAAAAGAGATTTTGAAACTTGTGCTTTGCACAAGTTGGGT
GGACACAGGTGGTGCATGGCTGTCGTCAGCTCGTGTCGTGAGATGTTGGGTTAAGTCCCGCAACGAGCGCAACCCTTGTTTTTAGTTGCTTTTA
AAATATACAATTTAAAAGGAATGCGAAGCATAACTTCGCATACTTTAGTATGTGAAGGAAACTACAAAGACTGCCGGTGATAAGCCGGAGGAAG
GTGAGGATGACGTCAAGTCAGCATGCCCCTTATGCCCTGGGCGACACACGTGCTACAATGGCCAGGACAATGAGATGCTACCTCGCGAGAGCAA
GCTAACCTCAAAAACCTGGTCTCAGTTCGGATTGCAGGCTGCAACTCGCCTGCATGAAGTCGGAATCGCTAGTAATCGCTGGTCAGCCATACAG
CGGTGAATACGTTCCCGGGCCTTGTACACACCGCCCGTCACACTACCAGAGTCGGCTTGGCCCAAAGTCGTAACTCTAACCTTCGGGAAGAGAG
CGCCTAAGGCAGGCTGGTAATGAGAGTGAAGTCGTAACAAGGTAGCCGTACTGGAAGGTGCGGCTGGATCACCTCCTTA

>ENA|AJ400848.1:134008..135498:rRNA|AJ400848.1:134008..135498:rRNA.1 Spinacia oleracea
(spinach) 16S ribosomal RNA
TCTCATGGAGAGTTCGATCCTGGCTCAGGATGAACGCTGGCGGCATGCTTAACACATGCAAGTCGGACGGGAAGTGGTGTTTCCAGTGGCGGAC
GGGTGAGTAACGCGTAAGAACCTGCCCTTGGGAGGGGAACAACAGCTGGAAACGGCTGCTAATACCCCGTAGGCTGAGAAGCAAAAGGAGGAAT
CCGCCCGAGGAGGGGCTCGCGTCTGATTAGCTAGTTGGTGAGGTAATAGCTTACCAAGGCGATGATCAGTAGCTGGTCCGAGAGGATGATCAGC

CACACTGGGACTGAGACACGGCCCAGACTCCTACGGGAGGCAGCAGTGGGGAATTTTCCGCAATGGGCGAAAGCCTGACGGAGCAATGCCGCGT
GGAGGCAGAAGGCCCACGGGTCGTGAACTTCTTTTCCCGGAGAAGAAGCAATGACGGTATCCGGGGAATAAGCATCGGCTAACTCTGTGCCAGC
AGCCGCGGTAAGACAGAGGATGCAAGCGTTATCCGGAATGATTGGGCGTAAAGCGTCTGTAGGTGGCTTTTTAAGTCCGCCGTCAAATCCCAGG
GCTCAACCCTGGACAGGCGGTGGAAACTACCAAGCTGGAGTACGGTAGGGGCAGAGGGAATTTCCGGTGGAGCGGTGAAATGCGTAGAGATCGG
AAAGAACACCAACGGCGAAAGCACTCTGCTGGGCCGACACTGACACTGAGAGACGAAAGCTAGGGGAGCGAATGGGATTAGATACCCCAGTAGT
CCTAGCCGTAAACGATGGATACTAGGCGCTGTGCGTATCGACCCGTGCAGTGTTGTAGCTAACGCGTTAAGTATCCCGCCTGGGGAGTACGTTC
GCAAGAATGAAACTCAAAGGAATTGACGGGGGCCCGCACAAGCGGTGGAGCATGTGGTTTAATTCGATGCAAAGCGAAGAACCTTACCAGGGCT
TGACATGCCGCGAATCCTCTTGAAAGAGAGGGGTGCCTTCGGGAACGCGGACACAGGTGGTGCATGGCTGTCGTCAGCTCGTGCCGTAAGGTGT
TGGGTTAAGTCCCGCAACGAGCGCAACCCTCGTGTTTAGTTGCCAACGTTGAGTTTGGAACCCTGAACAGACTGCCGGTGATAAGCCGGAGGAA
GGTGAGGATGACGTCAAGTCATCATGCCCCTTATGCCCTGGGCGACACACGTGCTACAATGGCCGGGACAAAGGGTCGCGATCCCGCGAGGGTG
AGCTAACCCCAAAAACCCGTCCTCAGTTCGGATTGCAGGCTGCAACTCGCCTGCATGAAGCCGGAATCGCTAGTAATCGCCGGTCAGCCATACG
GCGGTGAATTCGTTCCCGGGCCTTGTACACACCGCCCGTCACACTATGGGAGCTGGCCATGCCCGAAGTCGTTACCTTAACCGCAAGGAGGGGG
ATGCCGAAGGCAGGGCTAGTGACTGGAGTGAAGTCGTAACAAGGTAGCCGTACTGGAAGGTGCGGCTGGATCACCTCCTTT

>ENA|M82900|M82900.1 Nicotiana plumbaginifolia 16S rRNA gene.
TCTCATGGAGAGTTCGATCCTGGCTCAGGATGAACGCTGGCGGCATGCTTAACACATGCAAGTCGGACGGGAAGTGGTGTTTCCAGTGGCGGAC
GGGTGAGTAACGCGTAAGAACCTGCCCTTGGGAGGGGAACAACAGCTGGAAACGGCTGCTAATACCCCGTAGGCTGAGGAGCAAAAGGAGGAAT
CCGCCCGAGGAGGGGCTCGCGTCTGATTAGCTAGTTGGTGAGGCAATAGCTTACCAAGGCGATGATCAGTAGCTGGTCCGAGAGGATGATCAGC
CACACTGGGACTGAGACACGGCCCAGACTCCTACGGGAGGCAGCAGTGGGGAATTTTCCGCAATGGGCGAAAGCCTGACGGAGCAATGCCGCGT
GGAGGTAGAAGGCCCACGGGTCGTGAACTTCTTTTCCCGGAGAAGAAGCAATGACGGTATCTGGGGAATAAGCATCGGCTAACTCTGTGCCAGC
AGCCGCGGTAATACAGAGGATGCAAGCGTTATCCGGAATGATTGGGCGTAAAGCGTCTGTAGGTGGCTTTTTAAGTCCGCCGTCAAATCCCAGG
GCTCAACCCTGGACAGGCGGTGGAAACTACCAAGCTGGAGTACGGTAGGGGCAGAGGGAATTTCCGGTGGAGCGGTGAAATGCGTAGAGATCGG
AAAGAACACCAACGGCGAAAGCACTCTGCTGGGCCGACACTGACACTGAGAGACGAAAGCTAGGGGAGCGAATGGGATTAGATACCCCAGTAGT
CCTAGCCGTAAACGATGGATACTAGGCGCTGTGCGTATCGACCCGTGCAGTGCTGTAGCTAACGCGTTAAGTATCCCGCCTGGGGAGTACGTTC
GCAAGAATGAAACTCAAAGGAATTGACGGGGGCCCGCACAAGCGGTGGAGCATGTGGTTTAATTCGATGCAAAGCGAAGAACCTTACCAGGGCT
TGACATGCCGCGAATCCTCTTGAAAGAGAGGGGTGCCTTCGGGAACGCGGACACAGGTGGTGCATGGCTGTCGTCAGCTCGTGCCGTAAGGTGT
TGGGTTAAGTCCCGCAACGAGCGCAACCCTCGTGTTTAGTTGCCATCGTTGAGTTTGGAACCCTGAACAGACTGCCGGTGATAAGCCGGAGGAA
GGTGAGGATGACGTCAAGTCATCATGCCCCTTATGCCCTGGGCGACACACGTGCTACAATGGCCGGGACAAAGGGTCGCGATCCCGCGAGGTGA
GCTAACCCCAAAAACCCGTCCTCAGTTCGGATTGCAGGCTGCAACTCGCCTGCATGAAGCCGGAATCGCTAGTAATCGCCGGTCAGCCATACGG
CGGTGAATTCGTTCCCGGGCCTTGTACACACCGCCCGTCACACTATGGGAGCTGGCCATGCCCGAAGTCGTTACCTTAACCGCAAGGAGGGGGA
TGCCGAAGGCAGGGCTAGTGACTGGAGTGAAGTCGTAACAAGGTAGCCGTACTGGAAGGTGCGGCTGGATCACCTCCTTT

>ENA|AP000423.1:101012..102502:rRNA|AP000423.1:101012..102502:rRNA.1 Arabidopsis thaliana (thale cress) 16S ribosomal RNA
TCTCATGGAGAGTTCGATCCTGGCTCAGGATGAACGCTGGCGGCATGCTTAACACATGCAAGTCGGACGGGAAGTGGTGTTTCCAGTGGCGGAC
GGGTGAGTAACGCGTAAGAACCTGCCCTTGGGAGGGGAACAACAGCTGGAAACGGCTGCTAATACCCCGTAGGCTGAGGAGCAAAAGGAGGAAT
CCGCCCGAGGAGGGGCTCGCGTCTGATTAGCTAGTTGGTGAGGCAATAGCTTACCAAGGCGATGATCAGTAGCTGGTCCGAGAGGATGATCAGC
CACACTGGGACTGAGACACGGCCCAGACTCCTACGGGAGGCAGCAGTGGGGAATTTTCCGCAATGGGCGAAAGCCTGACGGAGCAATGCCGCGT
GGAGGTAGAAGGCCTACGGGTCCTGAACTTCTTTTCCCAGAGAAGAAGCAATGACGGTATCTGGGGAATAAGCATCGGCTAACTCTGTGCCAGC
AGCCGCGGTAATACAGAGGATGCAAGCGTTATCCGGAATGATTGGGCGTAAAGCGTCTGTAGGTGGCTTTTTAAGTCCGCCGTCAAATCCCAGG
GCTCAACCCTGGACAGGCGGTGGAAACTACCAAGCTTGAGTACGGTAGGGGCAGAGGGAATTTCCGGTGGAGCGGTGAAATGCGTAGAGATCGG
AAAGAACACCAACGGCGAAAGCACTCTGCTGGGCCGACACTGACACTGAGAGACGAAAGCTAGGGGAGCGAATGGGATTAGATACCCCAGTAGT
CCTAGCCGTAAACGATGGATACTAGGCGCTGTGCGTATCGACCCGTGCAGTGCTGTAGCTAACGCGTTAAGTATCCCGCCTGGGGAGTACGTTC
GCAAGAATGAAACTCAAAGGAATTGACGGGGGCCCGCACAAGCGGTGGAGCATGTGGTTTAATTCGATGCAAAGCGAAGAACCTTACCAGGGCT
TGACATGCCGCGAATCCTCTTGAAAGAGAGGGGTGCCTTCGGGAACGCGGACACAGGTGGTGCATGGCTGTCGTCAGCTCGTGCCGTAAGGTGT
TGGGTTAAGTCCCGCAACGAGCGCAACCCTCGTGTTTAGTTGCCACCGTTGAGTTTGGAACCCTGAACAGACTGCCGGTGATAAGCCGGAGGAA
GGTGAGGATGACGTCAAGTCATCATGCCCCTTATGCCCTGGGCGACACACGTGCTACAATGGCCGGGACAAAGGGTCGCGATCCCGCGAGGGTG
AGCTAACTCCAAAAACCCGTCCTCAGTTCGGATTGCAGGCTGCAACTCGCCTGCATGAAGCCGGAATCGCTAGTAATCGCCGGTCAGCCATACG
GCGGTGAATTCGTTCCCGGGCCTTGTACACACCGCCCGTCACACTATGGGAGCTGGCCATGCCCGAAGTCGTTACCTTAACCGCAAGGAGGGGG
GTGCCGAAGGCAGGGCTAGTGACTGGAGTGAAGTCGTAACAAGGTAGCCGTACTGGAAGGTGCGGCTGGATCACCTCCTTT

>ENA|AM087200.3:101968..103467:rRNA|AM087200.3:101968..103467:rRNA.3 Solanum lycopersicum (tomato) 16S ribosomal RNA
TCTCATGGAGAGTTCGATCCTGGCTCAGGATGAACGCTGGCGGCATGCTTAACACATGCAAGTCGGACGGGAAACACGGGAAACGGTGTTTCCA
GTGGCGGACGGGTGAGTAACGCGTAAGAACCTGCCCTTGGGAGGGGAACAACAGCTGGAAACGGCTGCTAATACCCCGTAGGCTGAGGAGCAAA
AGGAGGAATCCGCCCGAGGAGGGGCTCGCGTCTGATTAGCTAGTTGGTGAGGCAATAGCTTACCAAGGCGATGATCAGTAGCTGGTCCGAGAGG
ATGATCAGCCACACTGGGACTGAGACACGGCCCAGACTCCTACGGGAGGCAGCAGTGGGGAATTTTCCGCAATGGGCGAAAGCCTGACGGAGCA
ATGCCGCGTGGAGGTAGAAGGCCCACGGGTCGTGAACTTCTTTTCCCGGAGAAGAAGCAATGACGGTATCTGGGGAATAAGCATCGGCTAACTC
TGTGCCAGCAGCCGCGGTAATACAGAGGATGCAAGCGTTATCCGGAATGATTGGGCGTAAAGCGTCTGTAGGTGGCTTTTTAAGTCCGCCGTCA
AATCCCAGGGCTCAACCCTGGACAGGCGGTGGAAACTACCAAGCTGGAGTACGGTAGGGGCAGAGGGAATTTCCGGTGGAGCGGTGAAATGCGT
AGAGATCGGAAAGAACACCAACGGCGAAAGCACTCTGCTGGGCCGACACTGACACTGAGAGACGAAAGCTAGGGGAGCGAATGGGATTAGATAC
CCCAGTAGTCCTAGCCGTAAACGATGGATACTAGGCGCTGTGCGTATCGACCCGTGCAGTGCTGTAGCTAACGCGTTAAGTATCCCGCCTGGGG
AGTACGTTCGCAAGAATGAAACTCAAAGGAATTGACGGGGGCCCGCACAAGCGGTGGAGCATGTGGTTTAATTCGATGCAAAGCGAAGAACCTT
ACCAGGGCTTGACATGCCGCGAATCCTCTTGAAAGAGAGGGGTGCCTTCGGGAACGCGGACACAGGTGGTGCATGGCTGTCGTCAGCTCGTGCC
GTAAGGTGTTGGGTTAAGTCCCGCAACGAGCGCAACCCTCGTGTTTAGTTGCCATCGTTGAGTTTGGAACCCTGAACAGACTGCCGGTGATAAG
CCGGAGGAAGGTGAGGATGACGTCAAGTCATCATGCCCCTTATGCCCTGGGCGACACACGTGCTACAATGGCCGGGACAAAGGGTCGCGATCCC
GCGAGGGTGAGCTAACCCCAAAAACCCGTCCTCAGTTCGGATTGCAGGCTGCAACTCGCCTGCATGAAGCCGGAATCGCTAGTAATCGCCGGTC
AGCCATACGGCGGTGAATTCGTTCCCGGGCCTTGTACACACCGCCCGTCACACTATGGGAGCTGGCCATGCCCGAAGTCGTTACCTTAACCGCA
AGGAGGGGGATGCCGAAGGCAGGGCTAGTGACTGGAGTGAAGTCGTAACAAGGTAGCCGTACTGGAAGGTGCGGCTGGATCACCTCCTTT

>GU592207.1:122359..123849:rRNA, Oryza sativa Japonica Group (Japanese rice) 16S ribosomal RNA
tctcatggagagttcgatcctggctcaggatgaacgctggcggcatgcttaacacatgcaagtcgaacgggaagtggtgtttccagtggcgaac
gggtgagtaacgcgtaagaacctgcccttgggaggggaacaacaactggaaacggttgctaataccccgtaggctgaggagcaaaaggagaaat
ccgcccaaggaggggctcgcgtctgattagctagttggtgaggcaatagcttaccaaggcgatgatcagtagctggtccgagaggatgatcagc
cacactgggactgagacacggcccagactcctacgggaggcagcagtggggaattttccgcaatgggcgaaagcctgacggagcaatgccgcgt
ggaggtggaaggcccacgggtcgtcaacttcttttctcggagaagaaacaatgacggtatctgaggaataagcatcggctaactctgtgccagc
agccgcggtaagacagaggatgcaagcgttatccggaatgattgggcgtaaagcgtctgtaggtggcttttcaagtccgccgtcaaatcccagg

gctcaaccctggacaggcggtggaaactaccaagctggagtacggtaggggcagagggaatttccggtggagcggtgaaatgcattgagatcgg
aaagaacaccaacggcgaaagcactctgctgggccgacactgacactgagagacgaaagctaggggagcaaatgggattagagaccccagtagt
cctagccgtaaacgatggatactaggtgctgtgcgactcgacccgtgcagtgctgtagctaacgcgttaagtatcccgcctggggagtacgttc
gcaagaatgaaactcaaaggaattgacgggggcccgcacaagcggtggagcatgtggtttaattcgatgcaaagcgaagaaccttaccagggct
tgacatgccgcgaatcctcttgaaagagaggggtgccctcgggaacgcggacacaggtggtgcatggctgtcgtcagctcgtgccgtaaggtgt
tgggttaagtctcgcaacgagcgcaaccctcgtgtttagttgccactatgagtttggaaccctgaacagaccgccggtgttaagccggaggaag
gagaggatgaggccaagtcatcatgccccttatgccctgggcgacacacgtgctacaatgggcgggacaaagggtcgcgatctcgcgagggtga
gctaactccaaaaacccgtcctcagttcggattgcaggctgcaactcgcctgcatgaagcaggaatcgctagtaatcgccggtcagccatacgg
cggtgaatccgttcccgggccttgtacacaccgcccgtcacactataggagctggccatgtttgaagtcattacccttaaccgtaaggaggggg
atgcctaaggctaggcttgcgactggagtgaagtcgtaacaaggtagccgtactggaaggtgcggctggatcacctccttt

>ENA|AP005672.1:120253..121750:rRNA|AP005672.1:120253..121750:rRNA.1 Physcomitrella patens 16S rRNA
TCTCATGGAGAGTTTGATCCTGGCTCAGGATGAACGCTGGCGGCATGCTTAACACATGCAAGTCGGACGGAAAGCACCTTTTGGTGTTTTCAGT
GGCGGACGGGTGAGTAACGCGTAAGAACCTGCCCTTGGGAGGGGGACAACAGCTGGAAACGGTTGCTAATACCCCATAAGCTGAGGAGTAAAAG
GAGGAATCCGCCTAAGGAGGGGCTTGCGTCTGATTAGCTAGTTGGTGAGGCAATGGCTTACCAAGGCGACGATCAGTAGCTGGTCTGAGAGGAT
GATCAGCCACACTGGGACTGAGACACGGCCCAGACTCCTACGGGAGGCAGCAGTGGGGAATTTTCCGCAATGGGCGAAAGCCTGACGGAGCAAT
GCCGCGTGGAGGTAGAAGGCCCACGGGTCGTAAACTCCTTTTCTCAGAGAAGAAGCAATGACGGTATCTGAGGAATAAGCATCGGCTAACTCTG
TGCCAGCAGCCGCGGTAAGACAGAGGATGCAAGCGTTATCCGGAATGATTGGGCGTAAAGCGTCTGTAGGTGGCTTTTTAAGTCTGCTGTCAAA
TCCCAGGGCTCAACCCTGGACAGGCGGTGGAAACTACCAAGCTGGAGTACGGTAGGGGCAGAGGGAATTTCCGGTGGAGCGGTGAAATGCGTAG
AGATCGGAAAGAACACCAACGGCGAAAGCACTCTGCTGGGCCGACACTGACACTGAGAGACGAAAGCTAGGGGAGCGAATGGGATTAGATACCC
CAGTAGTCCTAGCCGTAAACGATGGATACTAAGCGCTGCGCGTATCGACCCGTGCAGTGCTGTAGCTAACGCGTTAAGTATCCCGCCTGGGGAG
TACGTTCGCAAGAATGAAACTCAAAGGAATTGACGGGGGCCCGCACAAGCGGTGGAGCATGTGGTTTAATTCGATGCAACGCGAAGAACCTTAC
CAGGGCTTGACATGCCGCGAATCCTTTTGAAAGAAAGGAGTGCCTTCGGGAACGCGGACACAGGTGGTGCATGGCTGTCGTCAGCTCGTGCCGT
AAGGTGTTGGGTTAAGTCCCGCAACGAGCGCAACCCTCGTGTTTAGTTGCCATCATTTAGTTTGGAACCCTAAACAGACTGCCGGTGATAAGCC
GGAGGAAGGTGAGGATGACGTCAAGTCAGCATGCCCCTTACGCCCTGGGCGACACACGTGCTACAATGGCCGGGACAAAGGGTCGCGACCCCGC
GAGGGCAAGCTAACCTCAAAAACCCGGCCTCAGTTCGGATTGCAGGCTGCAACTCGCCTGCATGAAGCCGGAATCGCTAGTAATCGCCGGTCAG
CCATACGGCGGTGAATCCGTTCCCGGGCCTTGTACACACCGCCCGTCACACTATGGGAGCTGGCCATGCCCGAAGTCGTTACCCTAACCGCAAG
GAGGAGGATGCCGAAGGCAGGGCTAGTGACTGGAGTGAAGTCGTAACAAGGTAGCCGTACTGGAAGGTGCGGCTGGATCACCTCCTTT

>ENA|CP000230.1:192528..194004:rRNA|CP000230.1:192528..194004:rRNA.1 Rhodospirillum rubrum ATCC 11170 16S ribosomal RNA
AGAGTTTGATCCTGGCTCAGGACGAACGCTGGCGGCAGGCCTAACACATGCAAGTCGAACGCATCCTTCGGGATGAGTGGCGCACGGGTGAGTA
ACACGTGGGAACGTACCTTGGAGTGCGGAATAATCTTTGGAAACGAGGACTAATACCGCATACGCCCTTAGGGGGAAAGATTTATCGCTCCAAG
ATCGGCCCGCGTCCGATTAGCTAGTTGGCGGGGTAATGGCCCACCAAGGCGACGATCGGTAGCTGGTCTGAGAGGATGGCCAGCCACACTGGGA
CTGAGACACGGCCCAGACTCCTACGGGAGGCAGCAGTGGGGAATATTGCGCAATGGGGGCAACCCTGACGCAGCCATGCCGCGTGAGTGAAGAA
GGCCTTCGGGTTGTAAAGCTCTTTCGGGTGTGAAGATGATGACGGTAACACCAGAAGAAGCCCCGGCTAACTTCGTGCCAGCAGCCGCGGTAAT
ACGAAGGGGGCAAGCGTTGTTCGGAATTACTGGGCGTAAAGAGCGCGTAGGCGGTCTGATTAGTCAGAGGTGAAATCCCAGAGCTCAACTTTGG
AACTGCCTTTGATACTGTTAGACTAGAATCCGTGAGAGGGTGGTGGAATTCCCAGTGTAGAGGTGAAATTCGTAGATATTGGGAGGAACACCAG
TGGCGAAGGCGGCCACCTGGCGCGGTATTGACGCTGAGGCGCGAAAGCGTGGGGAGCAAACAGGATTAGATACCCTGGTAGTCCACGCCGTAAA
CGATGAGTGCTAGATGTCGGGGTACATGTACCTCGGTGTCGCAGCTAACGCATTAAGCACTCCGCCTGGGGAGTACGGCCGCAAGGTTAAAACT
CAAAGGAATTGACGGGGGCCCGCACAAGCGGTGGAGCATGTGGTTTAATTCGAAGCAACGCGCAGAACCTTACCAGCCCTTGACATCCCGTGAC
ACTTCCAGAGATGGAAGGTTCCCTTCGGGGACACGGTGACAGGTGCTGCATGGCTGTCGTCAGCTCGTGTCGTGAGATGTTGGGTTAAGTCCCG
CAACGAGCGCAACCCTCATCTTCAGTTGCCAGCAAGTAACGTTGGGCACTCTGAAGAGACTGCCGGTGACAAGCCGGAGGAAGGTGGGGATGAC
GTCAAGTCCTCATGGCCCTTACGGGCTGGGCTACACACGTGCTACAATGGCGCCTACAATGGGCAGCGACCTCGCGAGGGGAAGCTAATCTCCA
AAAGGCGTCTCAGTTCGGATTGCACTCTGCAACTCGGGTGCATGAAGTCGGAATCGCTAGTAATCGTGGATCAGCATGCCACGGTGAATACGTT
CCCGGGCCTTGTACACACCGCCCGTCACACCATGGGAGTTGGTTCTACCCGAAGACGGTACGCTAACCGCAAGGAGGCAGCCGGCCACGGTAGG
GTCAGCGACTGGGGTGAAGTCGTAACAAGGTAGCCGTAGGGGAACCTGCGGCTGGATCACCTCCTTT
